# Losartan attenuates neuroinflammation and neuropathic pain in paclitaxel‐induced peripheral neuropathy

**DOI:** 10.1111/jcmm.15427

**Published:** 2020-06-02

**Authors:** Nataliia Kalynovska, Mickael Diallo, Dita Sotakova‐Kasparova, Jiri Palecek

**Affiliations:** ^1^ Department of Functional Morphology Institute of Physiology The Czech Academy of Sciences Prague Czech Republic

**Keywords:** losartan, macrophage, neuroinflammation, neuropathic pain, paclitaxel

## Abstract

Paclitaxel‐induced peripheral neuropathy (PIPN) is often associated with neuropathic pain and neuroinflammation in the central and peripheral nervous system. Antihypertensive drug losartan, an angiotensin II receptor type 1 (AT1R) blocker, was shown to have anti‐inflammatory and neuroprotective effects in disease models, predominantly via activation of peroxisome proliferator‐activated receptor gamma (PPARγ). Here, the effect of systemic losartan treatment (100 mg/kg/d) on mechanical allodynia and neuroinflammation was evaluated in rat PIPN model. The expression of pro‐inflammatory markers protein and mRNA levels in dorsal root ganglia (DRGs) and spinal cord dorsal horn (SCDH) were measured with Western blot, ELISA and qPCR 10 and 21 days after PIPN induction. Losartan treatment attenuated mechanical allodynia significantly. Paclitaxel induced overexpression of C‐C motif chemokine ligand 2 (CCL2), tumour necrosis alpha (TNFα) and interleukin‐6 (IL‐6) in DRGs, where the presence of macrophages was demonstrated. Neuroinflammatory changes in DRGs were accompanied with glial activation and pro‐nociceptive modulators production in SCDH. Losartan significantly attenuated paclitaxel‐induced neuroinflammatory changes and induced expression of pro‐resolving markers (Arginase 1 and IL‐10) indicating a possible shift in macrophage polarization. Considering the safety profile of losartan, acting also as partial PPARγ agonist, it may be considered as a novel treatment strategy for PIPN patients.

## 1 INTRODUCTION

Paclitaxel‐induced peripheral neuropathy (PIPN) and neuropathic pain are dose‐limiting side effects of anticancer treatment, which are poorly managed by existing treatments and usually result in premature cessation of chemotherapy treatment.^1^ Deeper understanding of the underlying mechanisms of PIPN could help the development of more effective anti‐neuropathic and pain treatments. Recently, growing body of evidence demonstrates that the activation and pro‐inflammatory activity of non‐neuronal cells, including invaded macrophages, play a pivotal role in the development and maintenance of neuropathic pain. Paclitaxel and lipopolysaccharide (LPS) share many common features, including structural analogy, and have similar effect on TLR4‐expressing cells.^2^ TLR4‐mediated signalling is implicated in pro‐nociceptive effect of paclitaxel on dorsal root ganglion (DRG) neurons, as well as in classical pro‐inflammatory M1‐like macrophage activation.^2^, ^3^, ^4^ Classically polarized macrophages (M1 type or LPS‐induced type) are characterized by CCR2 + profile, which is necessary for the CCL2‐oriented chemotaxis,^5^ production of TNFα and IL‐6, accompanied with high cytotoxic and phagocytic activity. Cytotoxic activity of M1 macrophages is dependent on the production of reactive oxygen species (ROS), mainly by nicotinamide adenine dinucleotide phosphate oxidase isoform (NOX2) and nitric oxide molecules (NO) by inducible nitric oxide synthase (iNOS).^6^, ^7^ A lysosomal protein CD68 is regularly used as a phagocytic marker of M1 macrophages.^8^, ^9^

Losartan, along with its initial angiotensin receptor type 1 (AT1R) blocking properties, is also a partial agonist of peroxisome proliferator‐activated receptor gamma (PPAR)‐γ^10^ and was shown to attenuate the development of different neurodegenerative disorders.^11^, ^12^ PPAR‐γ belongs to the nuclear receptor family of transcription factors, which primarily regulates the maintenance of metabolic homeostasis, including adipogenesis, inflammation and lipid metabolism.^13^ Additionally, PPARγ is the principal member of the PPAR family in promoting macrophage polarization shift towards pro‐resolving anti‐inflammatory M2‐like subtype.^14^, ^15^ Lately, the neuroprotective and anti‐inflammatory properties of PPARγ activation have been intensively studied on animal models of Parkinson's disease, ischaemic brain damage, and age‐related or LPS‐induced inflammation.^11^, ^16^, ^17^, ^18^ Those studies confirmed that protective properties of PPARγ agonists are connected with their ability to suppress the production of inflammatory factors and induce the expression of anti‐inflammatory and pro‐resolving proteins by macrophages, thus causing macrophage polarization towards the M2 phenotype.

In the present study, we have used behavioural, molecular and biochemical methods to evaluate the anti‐inflammatory and analgesic effects of systemic losartan treatment in the rat model of PIPN.

## 2 METHODS AND MATERIALS

All efforts were made to minimize animal suffering, to reduce the number of animals used and to utilize alternatives to in vivo techniques, if available. All experiments were approved by the local Institutional Animal Care and Use Committee and were consistent with the guidelines of the International Association for the Study of Pain, the National Institutes of Health Guide for the Care and Use of Laboratory Animals and the European Communities Council Directive of 24 November 1986(86/609/EEC).

### 2.1 Animals

Adult healthy male Wistar rats (250‐350 g) purchased from the local breeding facility (Institute of Physiology CAS) were used in this study. All measures were taken to minimize the number of animals used. Animals were kept on a 12‐hours light/dark cycle, adequate ventilation provided and room temperature maintained at approximately 22 ± 1°C, in clear plastic cages with soft bedding with standard pellet diet and water ad libitum. All behavioural experiments were carried out during the light phase of the cycle.

### 2.2 Experimental groups

Experimental rats were randomly assigned to 3 experimental groups: paclitaxel‐treated animals (PAC group, n = 30 in total), paclitaxel‐treated animals with losartan administration (LOS group, n = 28 in total) and vehicle‐administered animals (VEH group, n = 26). Baseline data for each group were obtained 1‐2 days before the start of PINP induction or vehicle administration. For behavioural, biochemical and molecular experiments, the analyses consisted in the comparison between the three groups for mechanical stimuli responses, protein level quantification and mRNA expression levels, respectively (n = 4‐9 for every experimental group).

### 2.3 Drug administration

#### 2.3.1 Paclitaxel administration

Paclitaxel was administered with i.p. injection (5 × 2 mg/kg, Mylan) on five alternate days. Rats in the control group received intraperitoneal injection of the vehicle (Kolliphor EL (Sigma‐Aldrich)/ethanol, 1:1) on the same 5 alternate days. On days 10 and 21 after the first injection, lumbar DRG and SCDH tissues were collected and further used for Western blot, qPCR and ELISA experiments.

#### 2.3.2 Losartan administration

Losartan was administered *per os* (*p.o*.), as was described previously.^9^ During the experiment, losartan (Lozap, Zentiva) was dissolved in the drinking water for the animals. The losartan solution was changed daily, and the amount consumed was registered. The average amount of losartan administered p.o. was 100 mg/kg of body weight daily. This dose level was chosen based on the results of our previous study^9^ and was designed to maintain the effective plasma levels of LOS metabolites.^19^, ^20^

### 2.4 Assessment of mechanical hypersensitivity

The baseline behavioural thresholds were measured before paclitaxel administration (referred as CTRL value), and next measurements were performed on days 7, 10, 14 and 21 after the first paclitaxel application. All behavioural measurements were done in a treatment blinded fashion. Mechanical paw withdrawal threshold (PWT) was assessed on hind paws using electronic dynamic plantar von Frey aesthesiometer (IITC Inc Life Science) as it was described before.^9^ The mechanical withdrawal threshold was the minimum pressure exerted (in grams) that triggered the paw withdrawal. Each stimulus was applied 4 times with 5 minutes between trials. Results from hind paws were averaged and SEMs calculated. CTRL values for PAC, LOS and VEH groups were 64.88 ± 1.27, 62.51 ± 1.45 and 65.49 ± 1.43 mN, respectively. In order to diminish the differences between the animals and groups, we expressed the experimental values as a percentage of the respective CTRL values in groups.

### 2.5 Western blot assay

#### 2.5.1 Tissue processing

Animals were deeply anesthetized with isofluranum (Forane, AbbVie). Briefly, L3‐L5 DRGs and L3‐L5 spinal cord sections were removed, the dorsal part of the spinal cord and the DRGs were frozen and stored at −80°C for later analysis.

#### 2.5.2 Protein sample preparation

Each sample was mechanically homogenized with hand‐held pellet pestle in CelLytic™ mammalian tissue lysis/extraction reagent (Sigma‐Aldrich) prepared with a protease inhibitor cocktail (w/v 1:10, Sigma‐Aldrich). The samples were then centrifuged (10 minutes, 2500 *g*, 4°C), and the supernatants were collected and centrifuged again (20 minutes, 16 500 *g*, 4°C). Supernatant protein concentration was determined by Bradford method using Bio‐Rad protein assay (Bio‐Rad), and the samples were diluted in a reducing sample buffer (1:1, v:v) to a range of concentration of 1 μg/μL.

#### 2.5.3 Immunoblotting

Protein samples were boiled for 3 minutes and separated by SDS‐PAGE 4%‐10% Bis acrylamide (Serva)‐Tricine (Sigma‐Aldrich) Gel with a sample volume of 20 μL/well. Proteins were then electrotransferred onto a nitrocellulose membrane (0.2 μm, Bio‐Rad). The membrane was first saturated by incubation in blocking solution (5% bovine serum albumin and 0.1% Tween‐20 in TBS) for 20 minutes and was then incubated overnight at 4°C with monoclonal mouse anti‐β‐actin (1:1000, loading control) and monoclonal mouse anti‐CD68 (1:1000); rabbit polyclonal β‐actin (1:500, loading control), rabbit polyclonal anti‐TNFα, rabbit polyclonal anti‐TNF‐R1, rabbit polyclonal anti‐CD11b, rabbit polyclonal anti‐GFAP, rabbit polyclonal anti‐CCL2, rabbit polyclonal anti‐CCR2 and rabbit polyclonal anti‐IL‐6 (1:000, 1:250, 1:500, 1:1000, 1:500, 1:250 and 1:250, respectively) in diluting solution. Blots were rinsed 3 times with 0.1% Tween‐20 in TBS and incubated for 90 minutes with fluorophore‐coupled goat anti‐mouse IR dye 800 and goat anti‐rabbit LiCor IRdye 680 (1:5000). Blots were rinsed 3 times with 0.1% Tween‐20 in TBS and then were scanned to reveal the protein bands with the Odyssey System Imager (Li‐Cor) coupled to acquisition software. Antibodies were provided by Exbio (anti‐CCL2, anti‐TNF‐R1, anti‐β‐actin), BioVision (anti‐CCR2), Neuromics (anti‐CD11b), Abcam (anti‐TNFα and anti‐IL‐6), Sigma (anti‐GFAP) and Li‐Cor (anti‐mouse, anti‐rabbit). The immunoreactivity of proteins of interest was compared with β‐actin immunoreactivity values controls and quantified based on scanned images of the blots, with Aida image analyser software (Aida™). For statistical analysis, values were obtained after first standardization of the raw values to β‐actin corresponding values and then after normalization using the VEH group spinal cord sample value as reference of 100%.

### 2.6 qPCR

At days 10 and 21 after the first injection of paclitaxel or vehicle, the animals were deeply anesthetized and tissues of interest were removed, frozen in liquid nitrogen and stored at −80°C. The total RNA from the spinal cord dorsal horn and DRGs was isolated with the commercially available kit RNeasy Mini (Qiagen) according to the manufacturer's protocol. Concentration and purity of total RNA were determined by measurements of the absorbance at 260 and 280 nm using a NanoDrop 1000 spectrophotometer (NanoDrop Technologies). Samples were treated with DNase (RNase‐free DNase set, Qiagen) to avoid the contamination with genomic DNA. cDNA was synthetized using ImProm‐II Reverse Transcription System (Promega Corporation). Quantitative PCR was carried out using a Viia 7 Real‐Time PCR System (Applied Biosystems), 5× Hot Firepol Probe QPCR Mix Plus (ROX) (Solis BioDyne) and premade TaqMan Assays (Thermo Fisher Scientific) specific for the studied transcript. The following assays were used: Arg1 (for Arginase 1, Cat. No. Rn00691090_m1), Actb (β‐actin, Rn00667869_m1), Ccl2 (CCL2, Rn00580555_m1), Ccr2 (CCR2, Rn01637698_s1), Fcgr1a (CD64, Rn01762682_m1), Cd68 (CD68, Rn01495634_g1), Gfap (GFAP, Rn00566603_m1), IL‐10 (IL‐10, Rn01483988_g1), IL‐6 (IL‐6, Rn01410330_m1), Nos2 (iNOS, Rn99999069_mH), Cybb (NOX2, Rn00576710_m1), Itgam (CD11b, Rn00709342_m1), Tnf (TNFα, Rn00562055_m1) and Pparg (PPARγ, Rn00440945_m1). β‐actin was chosen among four genes (β‐actin, GAPDH, HPRT, 18S) as a proper housekeeping gene for our experiments (Norm Finder software, MOMA). Fold differences of relative mRNA levels over vehicle control were calculated by 2^−ΔΔ^ *^C^* ^t^ method.^21^

### 2.7 Protein assay

The Arginase 1 protein levels in the DRG and SCDH tissues, collected at day 21 after the first paclitaxel injection, were measured by the ELISA kit according to the manufacturer's instructions using Rat Arg1 ELISA Kit (MyBioSource). Rats were deeply anesthetized with isofluranum; tissues were removed and immediately frozen in liquid nitrogen, then stored at −80°C. Prior to ELISA measurement, tissue samples were homogenized in PBS and processed for protein quantification using Pierce™ BCA Protein Assay Kit (Thermo Scientific).

### 2.8 Data analysis

For behavioural tests, the withdrawal responses to mechanical stimulation with the electronic von Frey were averaged for each animal. The mean values from all the animals in the group were averaged, and means ± SEM were calculated. Two‐way analysis of variance (ANOVA) followed by Holm‐Sidak post hoc test was used to assess statistical differences in PWT at different testing time points between the experimental groups. One‐way ANOVA followed by Holm‐Sidak post hoc test was used for the statistical comparison of protein and mRNA levels between the experimental groups. All values are expressed as means ± SEM. All statistical tests were performed using SigmaStat™ software.

## 3 RESULTS

### 3.1 Losartan treatment attenuates paclitaxel‐induced mechanical allodynia in rats

**Figure 1:**
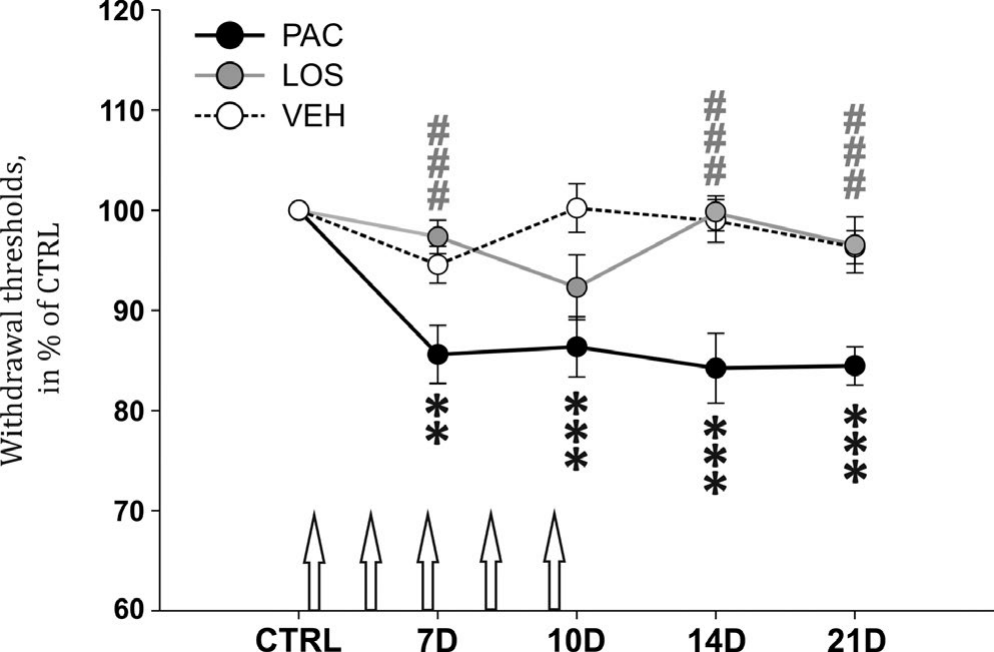
Systemic losartan treatment attenuates the development of mechanical allodynia, induced by repeated paclitaxel injections (arrows). Data are normalized to the CTRL value for each group and are presented as means ± SEM. Two‐way ANOVA followed by Holm‐Sidak post hoc test was used to assess the significance of statistical differences in PWT at different testing time points between the experimental groups. Asterisks indicate the significance of the comparisons between the experimental groups and corresponding values in VEH group (**P ≤ .01, ***P ≤ .001). Pound signs were used to depict the significant differences when compared to the PAC group (###*P* ≤ .001)

In our study, animals were treated with paclitaxel (5 × 2 mg/kg, i.p.) on 5 alternate days. Already on Day 7 after the first injection (after only 3 injections of paclitaxel), the PWT to mechanical stimuli in the PAC group (n = 30) significantly decreased to 85.6% (*P* < .01 when compared to VEH group (n = 26), Figure 1) and remained lower on Day 10 (86.4 ± 3.0%, *P* < .001 when compared to VEH group). This phase of PIPN may be considered as acute (while paclitaxel was still present in the tissues including DRGs). During the next phase (referred here as chronic), the PWT remained significantly decreased in the PAC group ‐ 84.2 ± 3.5% and 84.5 ± 1.9% of CTRL value on Days 14 and 21 respectively (*P* < .001, when compared to VEH group). Another experimental group of rats received systemic treatment with losartan simultaneously with the paclitaxel injections (from Day 1 until the end of the experiment, LOS group, n = 28). The behavioural changes observed in the LOS group seem to be two‐phasic. During the acute phase of PIPN on Day 7, the PWT to mechanical stimuli in this group was not different from the VEH group, but was different from the PAC group (97.3 ± 1.7%, *P* < .001). On Day 10, the PWT was lower (92.3 ± 3.3% of CTRL value at Day 10), but not significantly different from the CTRL value. During the chronic phase of PIPN, we observed a strong attenuation of the mechanical allodynia with the losartan treatment. On Days 14 and 21 after the first injection, the LOS group did not differ from the VEH group and the CTRL values (99.7 ± 1.8 and 96.5 ± 2.8% of the CTRL values) and the PWT were significantly different when compared with the PAC group ( *P* < .001 for both 10 and 21D).

### 3.2 Losartan has moderate effect on paclitaxel‐induced neuroinflammatory changes in the DRG and SCDH during the acute phase of PIPN

**Figure 2:**
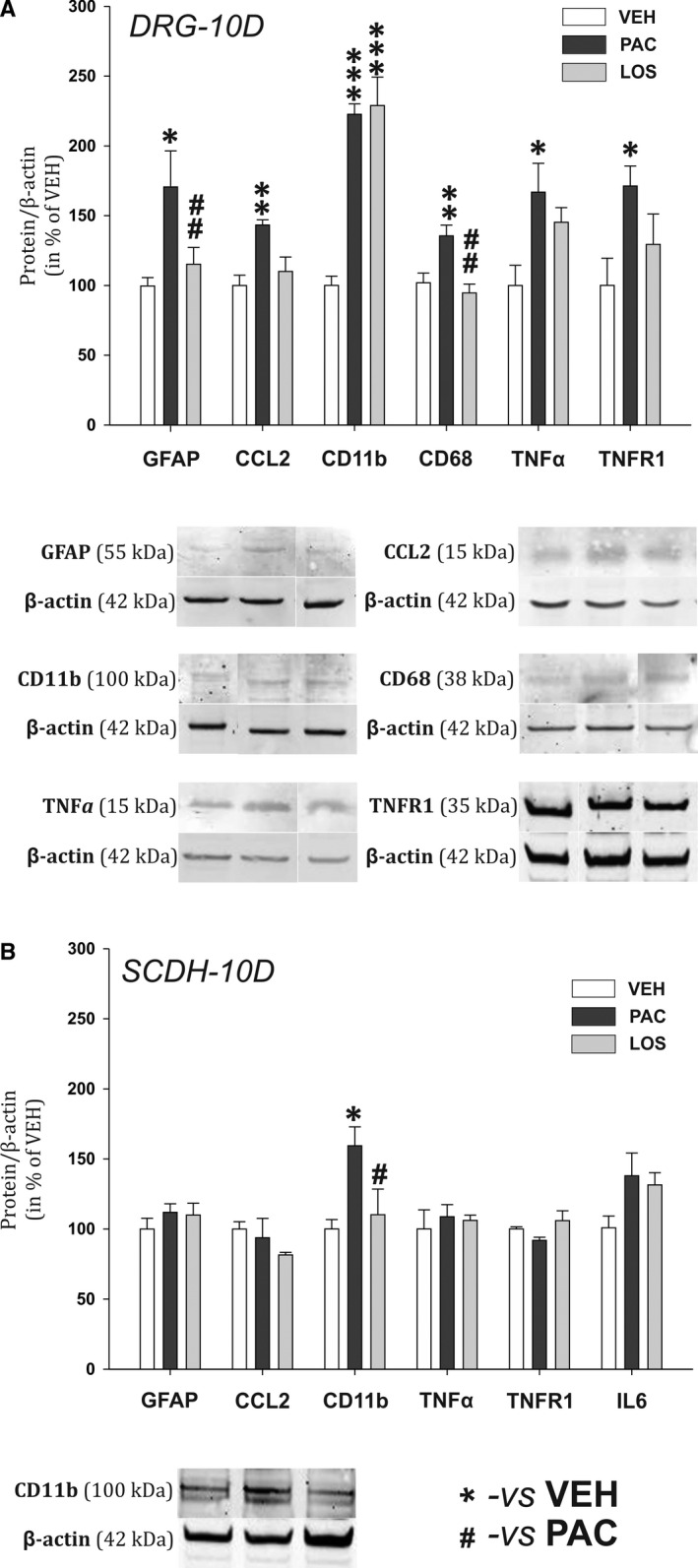
Relative protein expression levels in the DRG (A) and the SCDH (B) in experimental animals on Day 10 during the acute phase of PIPN. Representative Western blot captures for significantly affected proteins are presented below corresponding graphs. Data are normalized to β‐actin, standardized to the value in the VEH group and are presented as means ± SEM. One‐way ANOVA followed by Holm‐Sidak post hoc test was used for the statistical comparison. Asterisks indicate the significance of the comparisons to the VEH group (*P ≤ .05, **P ≤ .01, ***P ≤ .001). Pound signs were used to depict the significant differences when compared to the PAC group (#*P* ≤ .05, ##*P* ≤ .01)

**Figure 3:**
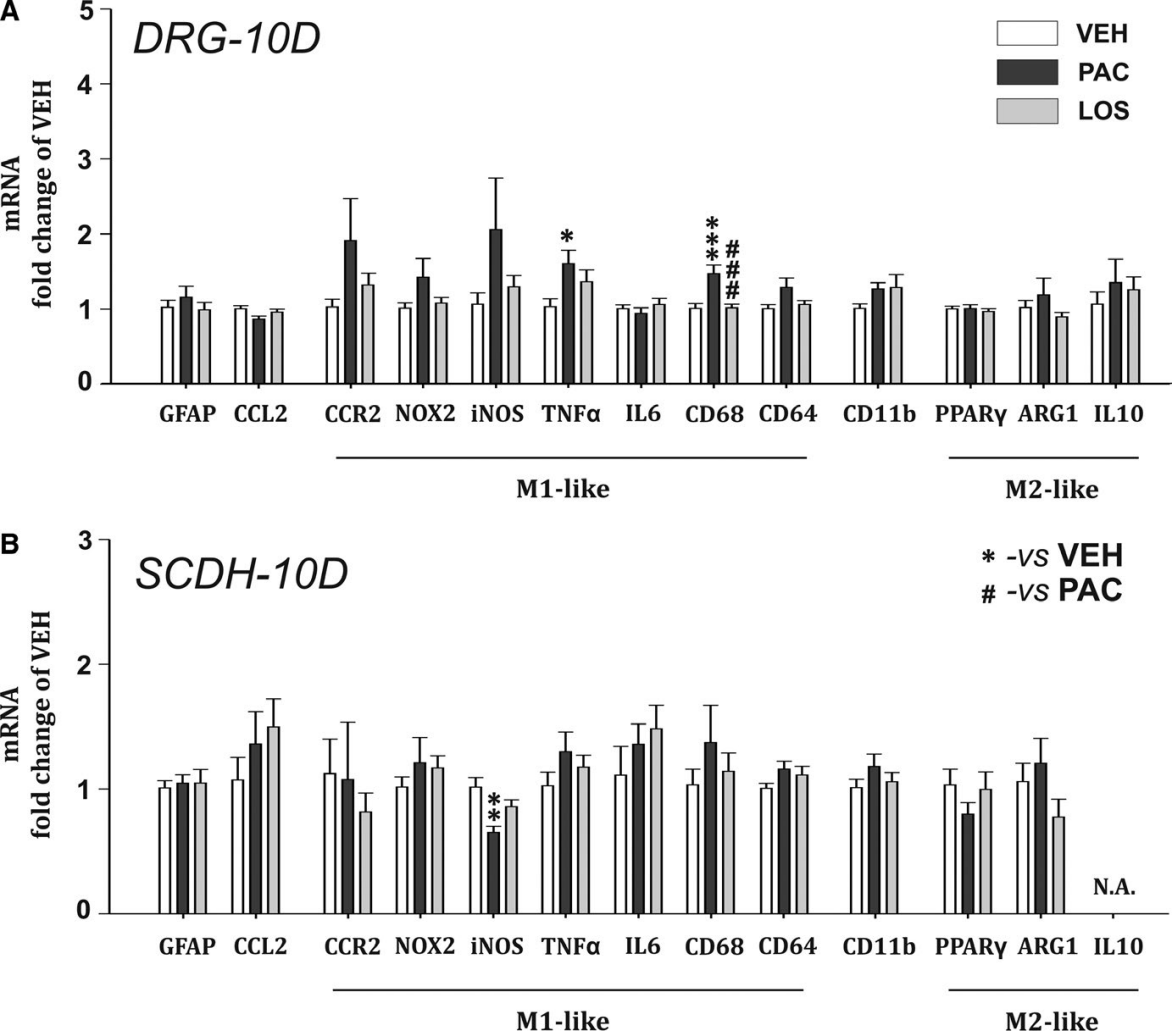
mRNA levels relative to the housekeeping gene β‐actin in the DRG (A) and the SCDH (B), measured during the acute phase of PIPN. VEH group (n = 6), PAC group (n = 5) and LOS group (n = 6). One‐way ANOVA followed by Holm‐Sidak post hoc test was used for the statistical comparison. Asterisks indicate the significance of the comparisons to the VEH group (*P ≤ .05, **P ≤ .01, ***P ≤ .001). Pound signs were used to depict the significant differences when compared to the PAC group (###*P* ≤ .001)

During the PIPN acute phase, expression of inflammatory proteins and specific mRNA levels measured on Day 10 suggest that neuroinflammatory changes were present predominantly in DRGs. Our results show a significant elevation of satellite glial cells (SGC) activation marker GFAP protein levels (*P* < .05 vs VEH group, n = 7 and 5 respectively, Figure 2A) as well as key inflammatory chemokine CCL2 (*P* < .01, n = 7 and 8). We observed also the presence of macrophage M1 cell line markers in the DRGs of paclitaxel‐treated animals. There were elevated levels of CD11b (*P* < .001, n = 4) and CD68 (*P* < .01, n = 5) proteins along with increased mRNA levels for CD68 (*P* < .001, n = 5, Figure 3A) and TNFα (*P* < .05) in the PAC group, when compared to the VEH group. The presence of activated macrophages in the PAC group was also confirmed by increased protein levels of TNFα (*P* < .05, n = 7, Figure 2A) as well as its specific receptor TNFR1 (*P* < .05, n = 5). Losartan treatment attenuated the activation of SGCs, as GFAP protein levels in the LOS group were significantly lower than in the PAC group (*P* < .01). The CCL2 chemokine increased expression was also prevented, as its protein levels in the LOS group did not differ from the VEH group. We did not observe elevation of M1 macrophage markers such as cytokine TNFα (n = 6), neither its mRNA, nor its receptor TNFR1 (n = 4), or phagocytosis marker CD68 (n = 5) in the DRGs of losartan treated rats. Moreover, losartan treatment significantly attenuated paclitaxel‐induced CD68 mRNA expression (*P* < .001 when compared with the PAC group, n = 6, Figure 3A). Surprisingly, we still observed a significant up‐regulation of CD11b protein overexpression in the LOS group when compared with the control group (*P* < .001, n = 4, Figure 2A).

In the spinal cord dorsal horn, we measured an increase of CD11b protein levels in the PAC group (Figure 2B), which was absent in the LOS group (*P* < .05, n = 6). We have observed a significant drop in iNOS mRNA expression in the PAC group (*P* < .01, n = 5, Figure 3B), while in the LOS group it was not significantly different. No other significant changes in mRNA or protein levels were detected of other inflammatory proteins in the spinal cord dorsal horn at this time point.

### 3.3 Losartan attenuates signs of paclitaxel‐induced neuroinflammation in the DRG and SCDH during the chronic phase

**Figure 4:**
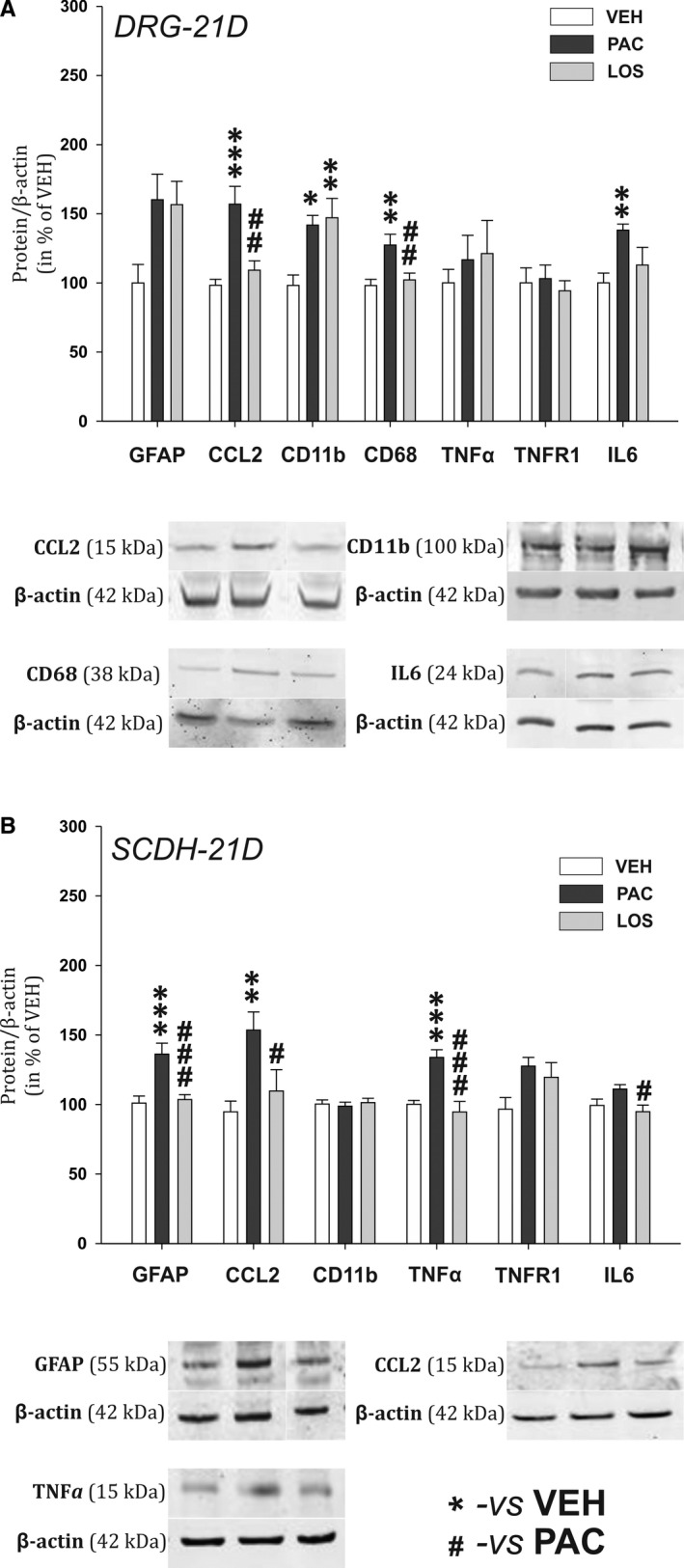
Relative protein expression levels in the DRG (A) and the SCDH (B) in experimental animals on Day 21 during the chronic phase of PIPN. Representative Western blot captures for significantly affected proteins are presented below corresponding graphs. Data are normalized to β‐actin, standardized to the value in the VEH group and are presented as means ± SEM. One‐way ANOVA followed by Holm‐Sidak post hoc test was used for the statistical comparison. Asterisks indicate the significance of the comparisons to the VEH group (*P ≤ .05, **P ≤ .01, ***P ≤ .001). Pound signs were used to depict the significant differences when compared to the PAC group (#*P* ≤ .05, ##*P* ≤ .01, ###*P* ≤ .001)

**Figure 5:**
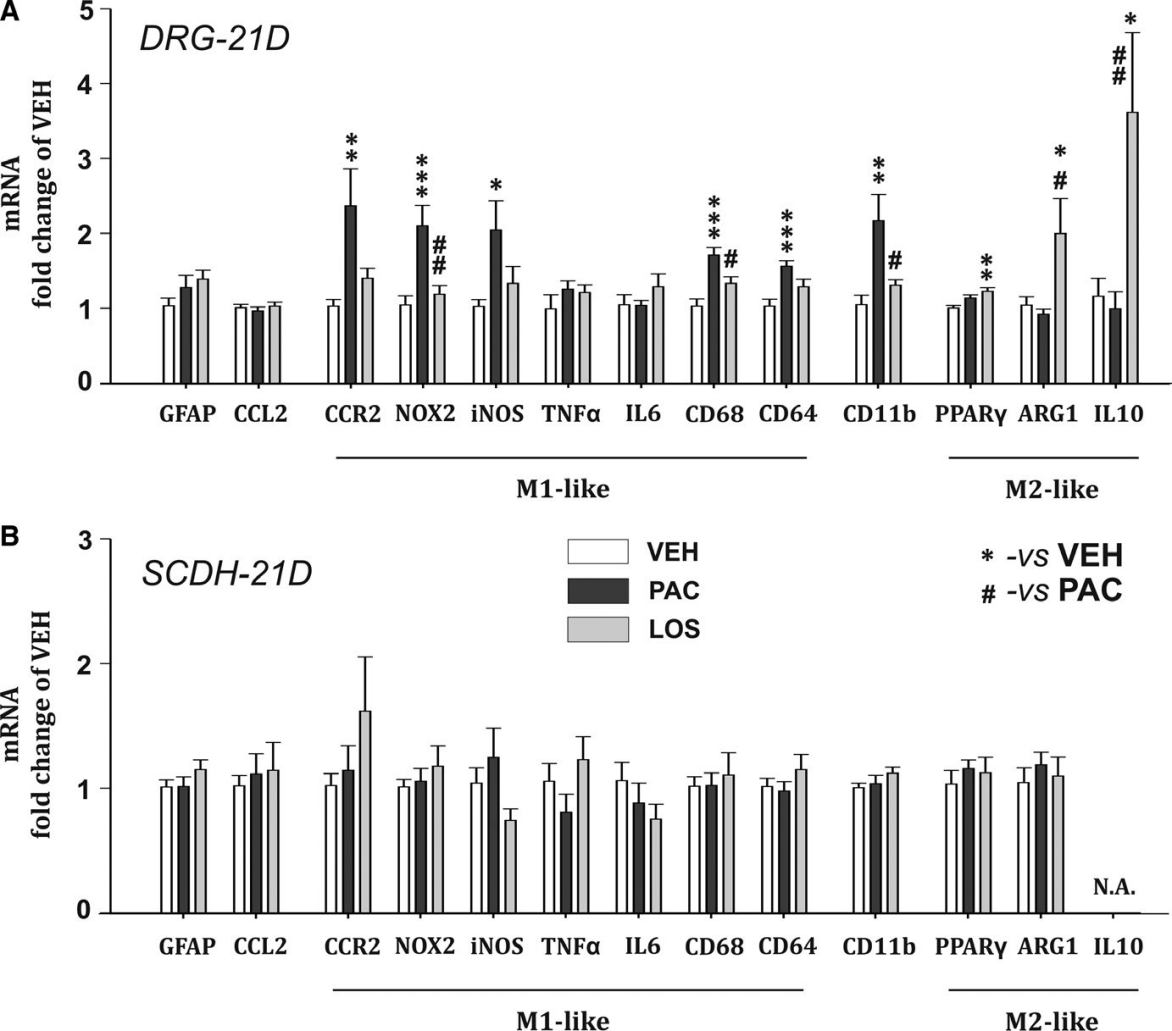
mRNA levels relative to the housekeeping gene β‐actin in the DRG (A) and the SCDH (B), measured during the chronic phase of PIPN. One‐way ANOVA followed by Holm‐Sidak post hoc test was used for the statistical comparison. Asterisks indicate the significance of the comparisons to the VEH group (*P ≤ .05, **P ≤ .01, ***P ≤ .001). Pound signs were used to depict the significant differences when compared to the PAC group (#*P* ≤ .05, ##*P* ≤ .01)

**Figure 6:**
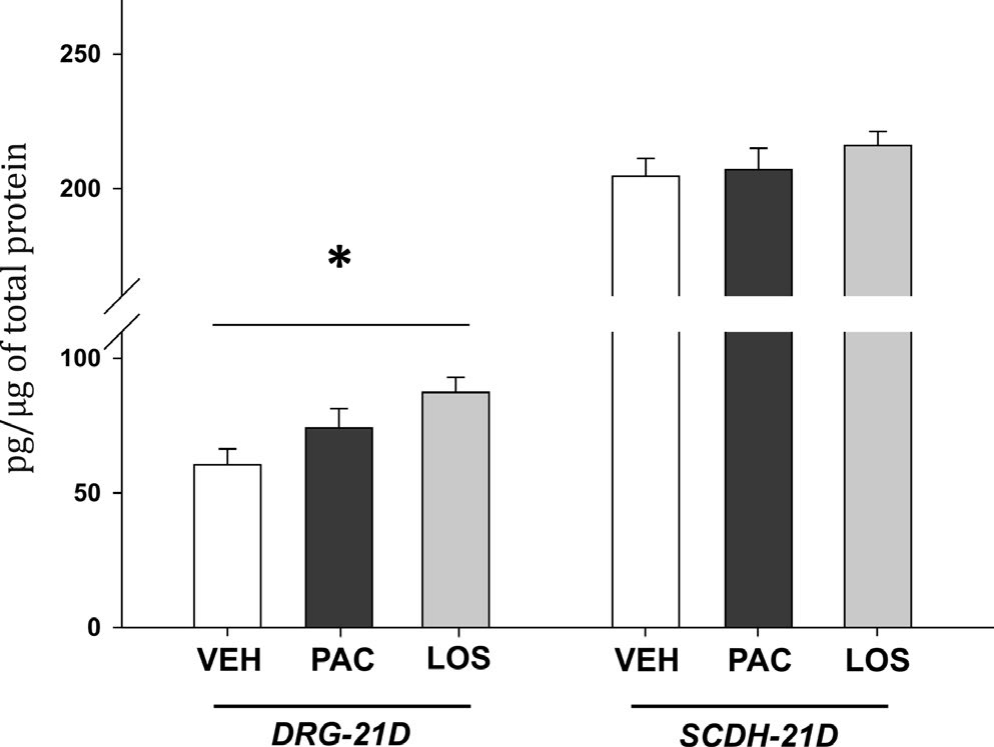
ELISA measurement of Arginase 1 protein levels in the DRG and the SCDH on Day 21 of PIPN. Data are presented as means ± SEM. One‐way ANOVA followed by Holm‐Sidak post hoc test was used for the statistical comparison. Asterisk indicate the significance of the comparison to the VEH group (*P ≤ .05)

In order to investigate neuroinflammatory changes during the chronic phase of PIPN, we have performed measurements of protein and mRNA levels at Day 21 after the start of the paclitaxel treatment. In the DRGs from the paclitaxel‐treated animals elevated levels of pro‐inflammatory proteins: chemokine CCL2 (*P* < .001, n = 8), cytokine IL‐6 (*P* < .01, n = 8) and macrophage markers CD11b (*P* < .05, n = 6) and CD68 (*P* < .01, n = 8, Figure 4A) were present. A significant increase in mRNA levels for specific M1 macrophage markers, CCR2 (*P* < .01), Nox2 (*P* < .001), iNOS (*P* < .05), CD64 (*P* < .001), CD68 (*P* < .001) and also for CD11b (*P* < .01), was also demonstrated when compared to the VEH group. In the LOS group, a significant decrease in the CCL2 protein levels (*P* < .01, n = 8) and prevention of IL‐6 overexpression in the DRGS was present when compared with the PAC group. While CD11b levels were still significantly elevated (*P* < .01 vs VEH group, n = 6), the paclitaxel‐induced protein overexpression of phagocytic marker CD68 was abolished by the losartan treatment (*P* < .01, n = 7). mRNA levels in DRGs of the LOS group (n = 7) indicated the attenuation of principal M1 macrophage markers up‐regulation when compared to the PAC group (n = 7)—Nox2 (*P* < .01, Figure 5A), CD11b (*P* < .05) and CD68 (*P* < .05). In contrast to the PAC group, the mRNA levels in the LOS group for CCR2, iNOS and CD64 did not differ from the VEH group values. In addition, a significant up‐regulation of the main M2 macrophage anti‐inflammatory product, IL‐10 mRNA expression (*P* < .05 vs VEH and *P* < .01 vs PAC groups), as well as ARG1 mRNA (*P* < .05 vs PAC and VEH groups) along with its protein product Arginase 1 (*P* < .05 vs VEH, n = 6, Figure 6) was present. Notably, PPARγ mRNA expression was also significantly increased in rats with the losartan treatment (*P* < .01 vs VEH group, n = 7).

In the spinal cord dorsal horn, we observed no signs of microglial activation (no significant increase in the CD11b protein expression), but robust astrocytosis, reflected in significant elevation of GFAP protein levels (*P* < .001, n = 7, Figure 4B) in the PAC group. Protein levels of key inflammatory modulators CCL2 and TNFα were also significantly increased (*P* < .01, n = 6, and *P* < .001, n = 8, respectively). Losartan treatment abolished the paclitaxel‐induced up‐regulation of GFAP (*P* < .001, n = 7) and chemokine CCL2 (*P* < .05, n = 7). TNFα and IL‐6 protein levels were also affected by losartan treatment and significantly decreased when compared to the PAC group (*P* < .001, n = 8, and *P* < .05, n = 8, respectively). However, we did not detect any changes in mRNA expression for these proteins at this time point (Figure 5B).

## 4 DISCUSSION

Paclitaxel treatment in rats leads to the development of peripheral neuropathy, characterized by mechanical allodynia occurring within days and lasting up to few weeks.^22^ In our study, we used this model to evaluate the effect of losartan treatment on the development of PIPN. Behavioural measurements confirmed the presence of neuropathic pain in the PAC group, and its significant attenuation in the LOS group at most time points, indicating pain resolving effect of the losartan treatment. Losartan also reduced neuroinflammation, demonstrated by significant changes in the expression of different markers.

Systemically applied paclitaxel crosses the blood‐brain barrier poorly. However, high concentrations persist in DRGs for up to 10 days and more after the last injection.^23^ Here, we consider 10 days of the paclitaxel treatment as an acute phase of PIPN, as paclitaxel was accumulating in DRGs reaching its maximal concentration after the last injection (Day 9). Behavioural changes observed during the acute phase were most probably related to paclitaxel‐associated acute pain syndrome.^24^, ^25^ During the next phase, referred in this study as chronic, the maintenance of neuropathic pain was probably mediated not only by direct paclitaxel effect and neuroinflammation in the DRGs but also by pathological changes on the central level, especially by neuroimmune modulation of synaptic transmission in the SCDH. Based on our results, mechanical allodynia was developing during the acute phase and reached its maximum in the chronic phase in the PAC group, while in the LOS group the behavioural changes seemed to be bi‐phasic. During the acute phase of PIPN, the reduction of PWT in the LOS group was attenuated on Day 7 compared to the PAC group and further reduced on Day 10. This corresponds with the low ability of losartan treatment to prevent acute paclitaxel‐induced changes in the DRGs.^25^ This may be also a reason for the reduction on Day 10 when the concentration of paclitaxel in the DRGs reached its maximum and the protective effect of losartan treatment was not enough to prevent the acute changes in the PWT. In spite of uncertain effect of losartan treatment over the acute phase, we observed robust prevention of mechanical allodynia in the LOS group during the chronic phase. Additionally, it was shown that losartan i.p. treatment was effective against PIPN‐induced behavioural and inflammatory changes after the PAC treatment was accomplished.^26^ Notably, administration of LOS to healthy rats does not significantly influence their thresholds to mechanical stimuli.^9^ Taken together, our data indicate preventive effect of losartan treatment on the neuropathic pain mainly during the chronic phase of PIPN, which is hypothesized to be mainly driven not by paclitaxel itself, but by secondary pathological neuroinflammatory changes.

During the acute phase of PIPN, the levels of inflammatory proteins expression and specific mRNA levels indicate that neuroinflammatory changes were present predominantly in the DRGs. Our results show significant elevation of GFAP and CCL2 protein levels in DRGs of PAC rats. CCL2 is the main chemoattractant agent attracting blood‐derived immune cells.^27^ Most anti‐PIPN strategies are focused on neuronal mechanisms, while growing number of studies indicate the essential role of macrophage infiltration in the PIPN development. Macrophage activation/infiltration profile is highly dependent on the drug, administration type, dosage and time schedule. For paclitaxel, macrophage activation in DRG may occur already 3 days after the treatment (for 4 × 2 mg/kg i.p.^28^ or 3 × 8 mg/kg i.p.^29^) or during the second week (for 2 × 16 mg/kg i.p.^30^ and 18 mg/kg iv^31^). Once in the tissues, monocyte‐derived macrophages respond to microenvironmental cues that determine whether they contribute to the establishment of local inflammatory response (M1‐like macrophages) or to its resolution (M2‐like subtype). We observed the presence of activated macrophages in the DRGs of PAC group already on Day 10, reflected in elevated levels of CD11b protein and CD68 protein and mRNA. The presence of activated macrophages was also confirmed by increased protein and mRNA levels of TNFα, the main cytokine product of M1 macrophages^32^ and its receptor TNFR1 protein. Losartan treatment abolished paclitaxel‐induced overexpression of GFAP, CCL2, CD68, TNFα and TNFR1 proteins, as well as mRNA for CD68 and TNFα in the DRGs. On the contrary, CD11b protein levels were significantly increased in the LOS group in the same manner as in the PAC group. While CD68 is a lysosomal protein expressed in phagocyting M1 macrophages,^8^ CD11b is expressed by innate immune system cells including microglia, ‘resident’ and ‘inflammatory’ monocytes.^33^ Therefore, CD11b is a marker of both M1 and M2 macrophages. As the levels of CD68 in the LOS group were significantly lowered, we may hypothesize that the main effect of losartan treatment was in the suppression of macrophage phagocyting activity, but not in the prevention of macrophage migration into the DRGs. We also measured a significant paclitaxel‐induced microglia activation (CD11b protein) and iNOS mRNA decrease in SCDH that were abolished in the LOS group. Activated microglia produces NO and high concentrations of NO reciprocally reduce iNOS mRNA expression.^34^, ^35^ While several previous publications demonstrate the absence of spinal microglia activation in PIPN,^36^ recently, it was shown that microglial activation plays an important role in the development of acute pain syndrome after PAC treatment.^31^, ^37^ Our data suggest that anti‐inflammatory effect of losartan was observed not only on activated macrophages in the DRG, but also on microglia in the CNS (directly or secondary by attenuation of the pro‐inflammatory signal input from the DRG).

Losartan is an antihypertensive drug, which blocks AT1R. However, macrophages do not express AT1R. Notably, AT1R is present in endothelial cells and their role in BBB integrity has been widely discussed recently. Activation of AT1R enhances BBB permeability and application of AT1R antagonists prevented BBB disruption and suppressed inflammatory events in the CNS.^38^, ^39^ However, the main observed effect of losartan treatment was on the attenuation of phagocytic activity in invaded macrophages. Different properties of losartan metabolites, EXP3174 and EXP3179, may explain the effect on macrophages. After the administration, losartan is catabolized into EXP3174 and EXP3179 metabolites. EXP3174 is a proper AT1R blocker, acting as a competitive antagonist,^40^, ^41^ while EXP3179 does not interfere with angiotensin II binding sites,^42^ but mediates the activation of PPARγ receptors.^10^ Since PPARγ response elements were identified in the promoter region of Arg1 gene, one of the suggested mechanisms of PPARγ agonist‐induced macrophage polarization shift is direct up‐regulation of Arginase 1 along with down‐regulation of TNFα and NO expression by macrophages.^17^ We assume that the anti‐inflammatory effect of losartan treatment could be mediated mainly by its EXP3179 metabolite and PPARγ agonism.

Substantiation of our hypothesis was more evident during PIPN chronic phase. In the DRGs of the PAC animals on Day 21, we observed the presence of macrophages reflected in the overexpression of CD11b protein. Increased levels of CCL2 protein along with CD68, and macrophage pro‐inflammatory product IL‐6, were significantly attenuated in the LOS group, indicating anti‐inflammatory effect of the losartan. Paclitaxel treatment induced mRNA up‐regulation of markers indicating M1‐like macrophage presence (CCR2, CD68, CD11b, NOX2, iNOS, CD64). This up‐regulation was abolished with the losartan treatment. Our data suggest that paclitaxel induced the chemotactic invasion of CCR2 + monocyte subset (CD11b marker) cells towards the CCL2 chemokine in the DRGs. These cells most likely exhibited active phagocytosis (CD68) and production of ROS (NOX2) and NO (iNOS). CD64, a receptor for immunoglobulin G, is expressed in macrophages and is also up‐regulated in the nociceptive DRG neurons during the BBB dysfunction.^43^ Thus, the increase in CD64 mRNA expression in PAC group may reflect both M1 macrophage presence and DRG neuronal activation during PIPN chronic phase.

Losartan treatment resulted also in a robust increase in expression of M2‐like macrophage markers ARG1 and IL‐10, suggesting the macrophage polarization shift. IL‐10 is anti‐inflammatory cytokine produced by M2 macrophages. Its pro‐resolving properties include macrophage deactivation, characterized by inhibition of pro‐inflammatory cytokines production via destabilization of mRNA transcripts for TNF or IL‐1β^44^ and increased anti‐inflammatory cytokine production.^33^ Elevated spinal IL‐10 protein levels induce pain relief in neuropathic pain models including PIPN.^44^, ^45^ Additionally, the expression of Arginase 1 is widely viewed as a typical marker of M2 macrophages^32^, ^46^ and is also regulated by PPARγ. Elevation of PPARγ mRNA levels in the LOS group may indicate positive feedback loop^47^ and thus serve as a confirmation of the hypothesis that losartan acts through these receptors.

Losartan treatment prevented also paclitaxel‐induced spinal astrogliosis and the expression of CCL2 and TNFα. CCL2 and TNFα play an important role in central sensitization and pathological pain development through the modulation of synaptic transmission in the SCDH.^48^, ^49^ Decrease in CCL2 and TNFα levels in LOS group corresponds to the significant attenuation of increased mechanical sensitivity in the behavioural responses.

Neuropathic pain resolving and anti‐inflammatory effects of losartan treatment, demonstrated in this study, represent a potential target for the elaboration of novel treatment strategies for PIPN patients.

## CONFLICT OF INTEREST

The authors declare no conflict of interest.

## AUTHOR CONTRIBUTION

NK performed behavioural experiments, ELISA, statistical analysis and data interpretation, made figures and wrote the manuscript. MD performed Western blot experiments. DSK and NK performed qPCR. JP designed and supervised the study, participated in the preparation of manuscript.

## Data Availability

The data that support the findings of this study are available from the corresponding author upon reasonable request.
